# Patent Adaptation and the Construction of Special Rights: The Evolution of the EU’s Animal Variety Protection System and China’s Path Forward

**DOI:** 10.3390/ani16142154

**Published:** 2026-07-11

**Authors:** Xianjie Zhou, Wenfei Zhang, Zonghuan Zhang, Baoku Cheng

**Affiliations:** 1 School of Law, Nankai University, Tianjin 300350, China; 1120231092@mail.nankai.edu.cn (X.Z.); 990035@nankai.edu.cn (B.C.); 2 College of Humanities & Social Sciences, Huazhong Agricultural University, Wuhan 430070, China; zhangwenfei@mail.hzau.edu.cn

**Keywords:** animal varieties, patent law, biotechnology governance, ethical review, protection of distinctiveness

## Abstract

Innovation in animal breeding is shifting from traditional phenotypic selection towards a biotechnology-intensive paradigm centred on genetic engineering, necessitating robust intellectual property protection. Unlike for plants, no unified international protection system exists for animal varieties. The European Union has established a distinctive framework for the patent protection of animal varieties through the application of exclusion rules, judicial interpretations, and ethical review mechanisms. An examination of its historical evolution reveals three distinct phases: initiation, expansion, and retrenchment. The EU’s approach reflects an ongoing effort to reconcile the imperatives of biotechnological innovation, the safeguarding of agricultural public resources, and ethical legitimacy. Nevertheless, interpretive divergences have persistently blurred the distinction between “technical processes” and “essentially biological processes,” while the shift in ethical review from a utilitarian balancing test to a value-based prohibition model has further diminished the system’s predictability. Drawing lessons from the EU experience and considering China’s imperative to revitalise its seed industry, this paper proposes that China should not simply expand the scope of patent protection. Rather, inspired by the plant variety rights system, China should consider establishing a sui generis animal variety rights regime. Concurrently, differentiated authorization criteria should be formulated, with clearly defined ethical and ecological boundaries and explicit limitations on rights, so as to strike a sustainable balance among innovation incentives, the public interest, and ethical considerations.

## Simple Summary

With the breakthrough of biotechnologies such as gene editing, animal breeding is transforming from a traditional agricultural activity into a high-tech industry. However, the intellectual property protection of animal varieties remains an institutional gap worldwide. This issue bears directly upon the incentives for breeding innovation, the security of germplasm resources, and the international competitiveness of the seed industry. The European Union, as the first jurisdiction to explore animal patent protection, has developed a distinctive framework for the patent protection of animal varieties by adapting the exclusion rules of the European Patent Convention. Yet, over three decades of institutional evolution have revealed the structural limitations of the patent system in protecting breeding achievements, as well as the predictability problems arising from embedding ethical review into patent procedures. This study systematically reviews the EU experience with a view to providing an institutional reference for China’s ongoing strategy of seed industry revitalisation. It finds that China should not simply expand the scope of patent protection. Rather, drawing on the experience of plant variety rights, China may establish a new animal variety rights system that is coordinated with patents for breeding technologies, so as to strike a balance among innovation incentives, the public interest, and ethical considerations.

## 1. Introduction

### 1.1. Background

Since the rise in agrarian civilization, animals have been domesticated and genetically improved through crossbreeding, selective breeding, and other techniques, giving rise to a multitude of varieties that serve agricultural production and daily life. For a long time, animal breeding relied on phenotypic observation and empirical experience, remaining a traditional agricultural activity driven by practical needs. In recent years, breakthroughs in gene-editing technologies such as CRISPR/Cas9 have fundamentally reshaped the technological paradigm of animal breeding. Modern breeding is no longer confined to phenotypic selection or traditional crossbreeding, but relies on genome engineering, marker-assisted selection, and data-driven precision breeding. In this context, animal varieties are transforming from traditional agricultural resources into biotechnological assets of strategic importance. These assets exhibit new characteristics, including technological intensity, substantial genetic modification, and considerable commercial value.

In recent years, advances in breeding technology are challenging the existing intellectual property regime. Article 27(3)(b) of the *TRIPS Agreement* imposes an obligation on Members to protect plant varieties, but contains no express provision for animal varieties and allows Members to exclude them from patentability, leading to considerable divergence in national policy positions. Currently, most jurisdictions, including China, primarily rely on indirect protection mechanisms, such as patents on breeding methods, administrative approvals, or trade secrets, rather than establishing animal varieties as subject matter of intellectual property protection.

The European Union is one of the jurisdictions with the most complex and extensive practice in the protection of animal varieties. Through the interaction of the *European Patent Convention (EPC)*, the *Directive on the Legal Protection of Biotechnological Inventions* (Directive 98/44/EC, hereinafter ‘*the Directive*’), and the case law of the European Patent Office (EPO), the EU has gradually established a distinctive governance model that integrates patent exclusion rules, technical exception clauses, and ethical review mechanisms. However, EU practice also clearly highlights the inherent tensions among technological innovation, the public interest in agriculture, and bioethical governance. While the patent system is more appropriate for protecting upstream technological inventions, its effectiveness remains insufficient when applied to animal varieties that exhibit both biological characteristics and ethical sensitivity. This paper systematically analyses the evolution of the EU’s patent protection system for animal varieties and, on that basis, explores the implications for China’s evolving regulatory framework for animal breeding.

### 1.2. Literature Review

The rapid development of animal genetic breeding technologies, particularly the widespread application of new genetic technologies (NGTs), has ushered animal breeding into a new era of precision and digitalisation. Against this backdrop, the definition of intellectual property rights and the legal protection of innovations in animal breeding have become critical issues in global intellectual property governance, agricultural modernisation, and biotechnology regulation. Existing research can be summarised into three main strands: the justification of patent protection for animal varieties; the shift in ethical review from separate regulation to institutional embedding; and the research lag and institutional gaps within the Chinese context.

#### 1.2.1. The Debate on the Justification of Animal Variety Patents

Early literature focused on the patentability of genetically modified animals and related biotechnological inventions. Temmerman analysed the institutional foundations of animal breeders’ rights from a comparative law perspective, pointing out that patent protection plays an indispensable role in attracting investment and driving technology commercialisation [1]. Gibbs et al. extended their analysis to institutional relationships, arguing that patents on biological breeding achievements are reshaping institutional relationships within the modern agricultural innovation system [2]. Schaeffer, using a dairy cattle breeding model, demonstrated the utility of animal-related patents in the commercialisation of breeding data [3]. Jozwiak, meanwhile, discussed the importance and scope of animal patent protection in developing countries [4]. Research at this stage corresponded to the early phase during which transgenic animal technology was transitioning from the laboratory to commercialisation, providing an initial framework for establishing the justification of animal intellectual property protection.

However, these studies have two limitations. First, almost without exception, they treat “patentability” as synonymous with “the necessity of protection”, failing to address whether the patent system is the optimal institutional arrangement for protecting animal breeding achievements. Second, most studies focus on the patentability of genetically modified animals and the scope of breeders’ rights, paying limited attention to the long-term evolution of governance structures for animal breeding innovation. In contrast to the relatively mature plant variety protection system under the UPOV framework, animal varieties still rely on fragmented mechanisms such as patents, trade secrets, contracts, and breeding registration, and have yet to form a comprehensive and dedicated system [5]. In recent years, new literature has begun to challenge the assumptions underlying the “patent default framework”. Cockbain and Sterckx argue that the dynamic interpretation of G 3/19 reveals the methodological risks inherent in protecting animal breeding achievements within the patent framework [6], yet few Chinese scholars have engaged with this line of inquiry.

#### 1.2.2. Ethical Issues: From Separate Regulation to Institutional Embedding

Early research treated ethical review and intellectual property protection as distinct regulatory spheres. As early as 1988, Dresser pointed out that animal biotechnology raises significant ethical concerns regarding animal suffering and biodiversity, while academic scrutiny of the embedding of ethical review within the operation of patent law has been insufficient [7]. Although the analysis by Das et al. covers both the intellectual property and ethical dimensions of animal biotechnology, it does not adequately explore the institutional interactions between the two [8]. In recent years, Case T 1553/22 (human–pig chimaera) has extended the focus of ethical controversy from “animal suffering” to “human dignity”. Barker and Brettell regard this case as a landmark application of the “purpose and object” interpretation of Article 53(a) of the *EPC* [9]; Boult and Wade point out that this decision establishes “human dignity” as an absolute red line, and that “significant therapeutic benefits” no longer constitute grounds for bypassing it [10].

More recently, scholars have increasingly called for incorporating animal welfare standards into the ethical review framework for legal regulation of animal breeding [11]. Empirical research indicates that public acceptance increases significantly when gene-editing technologies are directed at alleviating animal suffering rather than merely enhancing economic performance, and that animal welfare considerations are shifting from a marginal ethical issue to a core dimension of biotechnology governance. However, existing research has not yet addressed how the evolution of ethical review standards affects patent granting practices and, in turn, the predictability of the patent system. This gap is one of the issues that this paper seeks to address.

#### 1.2.3. The Lag in Chinese Research and Institutional Gaps

As a country with abundant animal genetic resources, China has, in recent years, witnessed the emergence of new varieties such as the thornless blunt-snout bream through the application of gene-editing technologies. Despite China’s recent policy initiative to “strengthen legislation in emerging fields”, academic attention remains disproportionately focused, with existing discussions on the protection of animal varieties scattered across the broader context of biodiversity conservation studies. Chinese scholar Zhang Zhongmin was among the first to explore fundamental issues regarding the patent protection of genetically modified animals, emphasising the importance of granting special rights in incentivising breeding innovation, however, his analysis remained confined to the question of “patentability” and did not address institutional design [12]. Liu Xuxia provided a comprehensive discussion of intellectual property rights in genetically modified biotechnology, but did not focus specifically on animal varieties [13]; Li Judan mentioned the necessity of legislation on animal variety rights, but did not elaborate [14]; Luo Li’s research on the protection of breeding innovation focused primarily on plants, with animals addressed only incidentally [15]. A survey by Shi et al. indicated that “the lack of specific protection for animal varieties” was listed as a major institutional obstacle for the breeding industry [16]. Wang’s empirical study on Chinese swine breeding enterprises reveals that small and medium-sized enterprises have virtually no access to intellectual property rights, and that the problem of institutional fragmentation is even more acute than in Europe [17]. Chen et al.’s empirical research indicates that current legal provisions contain institutional gaps in three areas—“unclear ownership, restricted transfer, and ambiguous distribution of rights and interests”—which directly affect investment in breeding [18]. However, most of these studies are largely confined to “problem description” and fail to explore in depth the institutional design of “solutions”.

#### 1.2.4. Shortcomings of Existing Research and the Present Study

A review of the aforementioned literature reveals significant shortcomings in existing research across three dimensions, which this paper seeks to address.

Firstly, there is a lack of theoretical analysis regarding the structural limitations of the patent system. Existing research largely treats patents as the default framework for the protection of intellectual property rights in animal breeding, focusing on “whether” and “how” to secure patents, yet rarely examines whether a structural tension exists between the patent system and the protection of animal breeding, or whether this tension can be bridged through incremental reforms such as judicial interpretations. Section 4 will argue that the threefold tension—regarding subject matter, patentability criteria, and scope of protection—constitutes a contradiction at the institutional-structural level that cannot be resolved through marginal adjustments.

Secondly, there is a lack of systematic examination of the institutional consequences of embedding ethical review within the patent process. While existing research acknowledges the importance of ethical review, it is often treated as an independent regulatory domain separate from the patent system. Section 3 of this paper will argue that the transformation of ethical review from a “utilitarian balancing” approach to a “value-based exclusion model” has not only altered the functioning of ethical review but has also had a significant impact on the predictability of the patent system.

Third, there is a lack of theoretical engagement with China’s protection framework. Existing research on China either remains at the level of problem description or borrows uncritically from foreign experiences, yet fails to address how institutional frameworks should be constructed to meet protection needs under the rigid constraints of Article 25 of China’s *Patent Law* and the practical demands of the breeding industry. Drawing on the experience of the European Union, this paper proposes a staggered integration scheme for the protection of technical patents and plant variety rights, and discusses specific design proposals.

### 1.3. Research Methodology

This paper employs literature analysis, normative analysis and comparative analysis in a progressive sequence: literature analysis to identify problems, normative analysis to examine causes, and comparative analysis to identify points of reference.

#### 1.3.1. Literature Analysis

Literature analysis systematically reviews relevant research on animal variety protection and the governance of biological breeding to construct a theoretical foundation and analytical framework. Literature sources include: Chinese literature sourced primarily from CNKI, focusing on domestic scholarship on “seed industry revitalisation” and intellectual property in animal breeding; English-language literature sourced primarily from Web of Science, covering EPO case law and relevant commentaries; and official EU documents, including the *EPC* Treaty, Directive 98/44/EC, and the case law of the EPO Enlarged Board of Appeal.

Based on this review, the analysis is conducted primarily at two main levels. First, the paper examines the technical evolution of emerging animal varieties and investigates how modern biotechnology has structurally reshaped traditional animal breeding models. This is followed by an analysis of the developmental trajectory and interpretive shifts in the EU’s animal variety patent protection system under Article 53(a) and (b) of the *EPC*, with particular attention to the repeated invocation of “dynamic interpretation” as an interpretive tool and its institutional costs. Second, in light of the needs of China’s biological breeding industry, this paper analyses the ethical implications embedded in Article 25 of The Patent Law—which excludes “animal varieties”—and the “public order and good morals” provision in the Examination Guidelines, as well as the reasons why China has struggled to address the protection of animal varieties through incremental patent reforms. This analysis provides a literature-based foundation for the subsequent discussion on establishing special rights of animal variety.

#### 1.3.2. Normative Analysis

This paper conducts a normative analysis of the relevant legal systems in the European Union and China, focusing on the normative interactions between statutory provisions, case law and implementing regulations. From the EU perspective, the primary legal sources include Article 53 of the *European Patent Convention*, Article 4 of Directive 98/44/EC, and case law such as G 2/12, G 2/13, G 3/19 and T 1553/22; from the Chinese perspective, the main legal instruments cover the *Patent Law*, the *Patent Examination Guidelines (2025 edition)* and the *Animal Husbandry Law*.

The normative analysis is structured around three levels. First, the distinction between “technical processes” and “essentially biological processes” under Article 53(b) of the *EPC* and the interpretative dilemmas arising therefrom. This part examines how the EPO examining divisions and the EPO Boards of Appeal (EBA) apply the requirement of “products exclusively obtained by essentially biological processes”, and dissects the ambiguities in demarcating technical processes from essentially biological processes in compound breeding scenarios. It further analyses the evolution and underlying rationale of the dynamic interpretation adopted in Rule 28(2) and Decision G 3/19, and reveals how administrative authorities counteract the erosion of legal certainty brought by judicial interpretations. Second, the paradigm shift in the ethical clause under Article 53(a) of the *EPC*. This section sorts out the transformation path from the utilitarian balancing test to the value exclusion model, and analyses the institutional costs to patent predictability caused by the shift in ethical review from “case-by-case balancing” to an “absolute exclusion zone”. Third, a normative investigation into China’s current legal regime for the protection of animal varieties. It focuses on an analysis of the rigid restriction and interpretative latitude of the “animal variety” exclusion stipulated in Article 25 of the Patent Law, as well as the functional misalignment between the existing administrative procedure of “variety approval” and intellectual property right confirmation. On this basis, it explores the normative grounds for establishing a specialised protection system for animal varieties.

#### 1.3.3. Comparative Analysis

The comparative analysis unfolds in three progressive stages, focusing primarily on the following three aspects to substantiate the central thesis that China cannot rely entirely on patents and must establish animal variety rights.

First, a comparison of institutional isomorphism—elaborating the internal logic underlying the EU’s role as the core reference model. By comparing the biopatent rules of China, the EU, and the US, this section clarifies the dual-track structure of “technical exclusions and ethical exclusions” shared by China and the EU. Drawing on this dimensional comparison, it argues that the EU’s dilemma of “incremental patent reform” carries significant cautionary value for China.

Second, a comparison of protection paradigms—taking the EU’s “incremental reform” pathway as an analytical sample. This section examines the convergent features in the evolution of biopatent rules between China and the EU, demonstrating that China cannot achieve the protection of animal varieties merely through adjustments to patent law interpretation. This provides empirical support for the core thesis of this paper that “China needs a protection framework transcending patent rules”.

Third, a detailed comparison of institutional structures—conducting a multi-level rule-matching analysis to assess the technical differences in the adaptability of DUS authorisation criteria to traditional breeding and genetically engineered animals, as well as a comparison of ethical review rules. The progressive application of these three methods forms the research framework of this paper: the literature review addresses the two gaps—the difficulty in applying patents and the lack of ethical constraints; the normative analysis examines the root causes of the EU’s institutional vacillation and the gaps in China’s regulatory framework; and the comparative analysis distils rules and approaches that can be adapted for China, thereby completing the research cycle.

## 2. The Evolutionary Logic and Regulatory Transformation of Animal Variety Patent Protection in the EU

The formation and evolution of animal variety patent protection in the EU are rooted in a multifaceted context characterised by the rapid iteration of modern biotechnology, the restructuring of the agricultural industry’s landscape of interests, and ongoing bioethical controversies. Throughout this process, the European Patent Office (EPO), through its ongoing interpretation and reconfiguration of relevant provisions of the *EPC*, has divided this evolution into three phases—breakthrough, expansion and restriction—in line with three major shifts in the EPO’s interpretation of Article 53 of the *EPC*.

### 2.1. From a Principle of Exclusion to the Breakthrough Phase of Protection for Genetically Modified Animals

From the perspective of institutional design, the European Union’s initial stance on animal varieties was one of exclusion in principle. Article 53(b) of the 1973 *EPC*, which succeeded the provisions of the 1963 Strasbourg Convention, explicitly excluded animal varieties and essential biological processes for the production of plants and animals from patent protection. This provision stemmed from traditional patent law theory, which was influenced by empiricism and had long upheld the principle that “living organisms are not patentable”, regarding organisms capable of independent biological activity as subjects unsuitable for inclusion in the patent system [19].

The application of genetic engineering technology has prompted innovators to seek to extend the scope of protection from technical solutions to animal varieties themselves. The “Harvard Oncomouse” case (T 19/90) marked a milestone in European animal patent law. In 1985, Harvard University filed a patent application with the EPO through DuPont; in 1989, the Examining Division directly rejected the application pursuant to Article 53(b) of the *EPC*; and in October 1990, the Technical Board of Appeal overturned this decision [20]. The core point of contention in this case centred on the interpretation of the scope of “animal varieties” under Article 53 of the EPC [21]. The applicant argued that the three official language versions of the *EPC* contained taxonomic differences regarding the scope of exclusion, with serious semantic conflicts existing between “animal varieties”, “races animates” and “Tierarten”. The Board of Appeal held that Harvard University’s patent application concerned the introduction of specific exogenous oncogene sequences, the technical feasibility of which spanned multiple species belonging to the highly general taxonomic categories of mammals and rodents, and thus did not fall within the exclusive scope of specific “varieties” [22]. On this basis, it was held that Article 53(b) of the *EPC* excludes specific, taxonomically low-level “animal varieties”, rather than “animals” in general. This logic was subsequently incorporated into *EPC* Rule 23c(b) and confirmed in the Novartis case (Case G 1/98): provided that the technical solution of the invention is not limited to a single plant or animal variety, the claims—even if they cover multiple varieties—do not fall within the scope of the exclusion under Article 53(b).

The EPO’s examination of the Tumour Mouse case underwent a shift from an initial outright rejection to a compromise granting of a patent; this shift was not a natural evolution of the internal logic of the law, but rather a passive compromise under external pressure, stemming primarily from three driving forces. First, technological competition and patent barriers between the US and Europe. In 1980, the US Supreme Court cleared the legal obstacles to patent protection for living organisms in the case of Diamond v. Chakrabarty, and in 1988 the USPTO granted the world’s first patent for a genetically modified mammal (US 4,736,866) [23]. By contrast, the EPO remained hesitant, constrained by the “public order and morality” and “exclusion of plant varieties” provisions of the *EPC*. European policymakers realised that if they continued to adhere to conservative examination standards, European research institutions and biotechnology companies would relocate to the United States, exposing the EU to the risks of capital flight and technological hollowing-out [24]. Secondly, there was pressure to comply with Article 27.3(b) of the *TRIPS Agreement*. The examination of the tumour-bearing mouse patent coincided closely with the GATT Uruguay Round negotiations. The *TRIPS Agreement* requires member states to provide patent protection for ‘microorganisms’ and ‘non-biological and microbial processes’, while the claims for the tumour-bearing mouse covered prior molecular manipulation and genetic recombination techniques; a complete rejection would have risked breaching compliance obligations under the *TRIPS Agreement* [25]. Thirdly, there was concerted lobbying by multinational pharmaceutical giants and venture capital firms. Biotechnology research and development is characterised by high investment, high risk and long development cycles. Companies such as DuPont had already invested substantial capital in the field of genetic engineering and, through industry organisations such as the European Biotechnology Industry Organisation (EuropaBio) and the Senior Advisory Group on Biotechnology (SAGB), exerted lobbying pressure on the European Commission and the European Patent Office [26]. At the same time, tumour-bearing mice, as living tools capable of reliably screening anti-cancer drugs, are highly recognised within the medical community; this medical value, which benefits humanity, provided the EPO with a legal basis.

In summary, the tumour-bearing mouse case is not an isolated incident, but rather a window of protection passively opened by the convergence of external pressures on Article 53 of the *EPC*; driven by both commercial interests and considerations of geo-industrial security, European regulators ultimately chose to compromise. This case established two core examination criteria: firstly, the “animal variety exclusion” under Article 53(b) was narrowly interpreted to apply only to “species and lower taxonomic units”; secondly, the Utilitarian Balancing Test established under Article 53(a). This technical definition of the scope of varieties, coupled with the ethical weighing of interests, has also led to institutional dilemmas for both aspects.

### 2.2. The Parallel Phase of Technical Solution Protection and the Expansion of Variety Protection

In 1988, the European Commission began drafting the *Directive on the Legal Protection of Biotechnological Inventions*, with the aim of harmonising the fragmented rules on biotechnology patents across the European Union. Article 4(1) of the *Directive*, adopted in 1998, reaffirmed the exclusion rule set out in Article 53 of the *EPC*, establishing the basic framework for the unpatentability of animal varieties; however, Article 4(3) stipulated that the exclusion from protection did not affect the patentability of inventions relating to microbiological or other technical processes, and Article 3(2) provided that biological material isolated from the natural environment by means of a technical process remained patentable. The Directive essentially constitutes a legislative endorsement of the examination framework established in Case T 19/90. The rationale behind the Directive stems from multiple policy considerations, including: preserving the public nature of traditional agricultural and breeding resources; preventing the excessive privatisation and monopolisation of breeding resources; providing necessary intellectual property incentives for modern biotechnologies such as genetic engineering; and maintaining the internal consistency of the intellectual property system to avoid overlap and conflict with plant variety rights.

However, subsequent institutional practice has been inconsistent; due to the consistency of legal reasoning, patent cases concerning plant varieties have directly driven the expansion of the scope of patent protection for animals. In the ‘Tomato II’ and “Cauliflower II” cases (G 2/12, G 2/13) [27], applicants attempted to circumvent method patents by claiming rights solely over end products possessing specific desirable traits. EBA adopted a dynamic interpretation approach to interpret Article 53(b) of the *EPC* broadly, holding that plants and animals obtained by means of a substantial biological process for the production of plants or animals are not excluded, and explicitly stating that this decision could be extended to apply to animals.

Placing G 2/12 and 13 within the interpretative history of Article 53(b) of the *EPC* reveals that, as neither the *EPC* nor *the Directive* clarified the scope of “plant and animal varieties”, T 356/93 (Tomato I) extended the scope of “varieties” to any plant population possessing identity; G 1/98 further excluded substantial biological processes and their products. However, the “product-by-implication exclusion” lacks a legal basis, and this line of interpretation is no longer tenable. Breeders may circumvent the exclusion by reformulating method claims as “method-defined products”; for example, by applying for patents on molecular markers for identifying specific traits (EP 1358356 A1) [28] or genotyping methods (EP 2597159 A1) [29]. Although the relevant patents cannot directly claim ownership of specific “animal varieties”, the right holder, by controlling key technical methods or genetic components, can still effectively govern the breeding practices and commercial exploitation of animal populations carrying specific traits [30]. In other words, G 2/12 essentially represents a reallocation of the scope of protection under the EBA: by removing plants and materials obtained through substantive biological methods from the scope of exclusion, it broadens the range of patentable subject-matter to include products, thereby amending G 1/98.

From a technical and industrial perspective, the amendment introduced by G 2/12 was necessary. When the *EPC* was drafted, substantial biological methods were limited to hybridisation or breeding; however, with the emergence of techniques such as molecular marker-assisted selection (MAS) and gene editing, it would be unreasonable to exclude related products merely because they originated from hybridisation or breeding. Furthermore, major powers in the plant science R&D sector, such as the Netherlands, Germany and the United Kingdom, exerted sustained pressure to prompt the EPO to make adjustments within the EU legal framework. From the perspective of external factors, as the EPO is a self-financing organisation, applying examination standards that are more stringent than those of the USPTO or JPO would lead to an outflow of capital and applications. This paper argues that Resolutions G 2/12 and G 2/13 are not merely interpretations of the *EPC*, but also implicitly assert the EPO’s institutional independence: the EPO is an independent system under the *EPC* Treaty, and even though all EU Member States are Contracting Parties to the *EPC*, the authority to interpret the *EPC* autonomously.

### 2.3. The Phase of Restriction and Dynamic Adaptation in Legislative and Administrative Responses

Following Decisions G 2/12 and 13, there was a surge in applications for patent protection for plants and animals. Statistics indicate that over 5000 applications were filed, of which more than 3000 were granted. Although no direct patents on animal varieties appeared in formal terms, patent protection not only extended to animals as “products” but also effectively brought the animal breeding process under control. This sparked strong concerns among Member State governments, the European Seed Association (ESA) and agricultural conservatives. Critics point out that this approach undermines the substantive effectiveness of the exclusion clauses and may lead to excessive control over basic agricultural resources and the upstream of the industrial chain, thereby damaging the innovation ecosystem of the breeding sector and farmers’ rights [31].

Just six months after the G 2/12 decision was issued, the EBA’s expansive interpretation prompted intervention by EU legislative and administrative bodies. The European Parliament adopted a non-legislative resolution expressing concern and calling for opposition to the patenting of conventional breeding products. Then, this Resolution formed the Council’s conclusion [32]. In 2016, the EPO Administrative Council adopted Resolution CA/D 6/1 almost unanimously to amend the *Implementing Regulations to the European Patent Convention*, adding a new Article 28(2) which explicitly stipulates that “pursuant to Article 53(b), a European patent shall not be granted for plants or animals obtained exclusively by means of a substantial biological process” [33]. This amendment directly overturns the EBA’s interpretation in cases G2/12 and G2/13, excluding from patent protection products obtained solely through hybridisation, selection and similar processes.

There are three driving factors behind this backlash. Externally, compliance pressures at Member State level are the primary driver. EU Member States are required to implement *the Directive*; if the scope of the EPO’s authorisation is broader than that of *the Directive*, Member States will find themselves in a conflict of interest. This was precisely the main reason why the European Commission issued an interpretative notice in November 2016 to urge the Contracting Parties to amend the Implementation Guidelines. Internally, the logical inconsistencies left by G 2/12 and 13 are difficult to reconcile; the distinction between methods and products in conventional breeding scenarios would effectively circumvent the exclusion provisions of the *EPC*, and the EPO has struggled to explain this legal loophole [34]. Although the Administrative Council, comprising representatives of the Contracting States, cannot directly overturn decisions issued by the EBA, its adoption of an amendment to Rule 28(2) of the Implementing Regulations to circumvent the EBA’s interpretation reflects the divergence between the administrative and judicial branches. At the technical and industrial levels, gene-editing technology had already begun to be applied by 2015. If conventional hybrid breeding were to be patentable, the boundary as to whether breeding following gene editing constitutes a product obtained by an essentially biological process would become blurred. Rule 28(2), which stipulates “exclusively obtained by an essentially biological process”, effectively excludes technical steps such as gene editing, thereby serving the interests of industrial sectors such as large seed companies.

However, the controversy surrounding Article 53(b) of the *EPC* has not subsided. In Case T 1063/18 in 2018, the EPO Technical Board of Appeal ruled, pursuant to Article 164(2) of the *EPC*, that Rule 28(2) contravened Article 53(b) of the *EPC*; this not only ran contrary to the political intent of the Administrative Council but also rendered the reasoning in Resolutions G 2/12 and G 2/13 internally inconsistent. The final compromise emerged in the 2020 “Pepper case” (Decision G 3/19):the EBA rejected the suggestion that “Rule 28(2) should be disregarded”, as well as the assertion that “G 2/12 had definitively resolved the issue of Article 53(b)”. While acknowledging the conclusions of G 2/12, it pointed out that the inclusion of Rule 28(2) had brought about a significant change in the legal facts on which G 2/12 was based [35]. The EBA reiterated the need for a “dynamic interpretation” to take full account of technological developments, legislative amendments and shifts in societal perceptions. Ultimately, Article 53(b), when interpreted dynamically, was extended to exclude “plants obtained entirely through substantial biological processes (the same legal principle applies to animals)”, while microbiological or other technical methods remained unaffected [36].

Following on from G 3/19, the EPO amended its *Examination Guidelines* in March 2021, emphasising that the examination must involve a specific analysis of the breeding process to determine whether human technical intervention has substantially altered that process. In March 2023, the EPO further amended the *Examination Guidelines*, emphasising that the criterion for assessing the degree of technical intervention is defined as playing a “decisive” role [37]. Consequently, the EU’s patent protection for animal varieties has undergone a significant shift; the core logic no longer rests on a simple distinction between “animal varieties” and “technical inventions”, but has shifted towards a substantive examination of the “degree of technical intervention”. This shift implies that the focus of examination has moved from the “classification of the subject-matter” (whether it constitutes a variety) to the “nature of the process” (whether the technical intervention reaches a decisive level), although the standard for a “decisive role” itself remains to be further clarified [38].

## 3. Analysis and Reflection on the EU System for the Patent Protection of Animal Varieties

Overall, the current protection of animal varieties in the EU primarily relies on a patent system that has been subject to special interpretation. This model offers several advantages: low operational costs, owing to its seamless integration with existing frameworks for the examination of biotechnological inventions and ethical assessment; strong internal coherence, as it enables the coverage of biotechnological inventions and the embedding of ethical review through patent adaptation; and a certain degree of safeguard for the public interest in agriculture, preventing excessive intellectual property protection from encroaching unduly upon agricultural production space. However, its limitations are equally pronounced:

### 3.1. Power Struggles over Interpretative Authority and Divergences in Protection Methods Among Multiple Stakeholders

The crux of the EU’s animal patent protection dilemma lies not in textual ambiguities surrounding Article 53(b) of the *EPC*, but in the overlapping and interweaving of the regulatory framework—which has resulted in the interpretation of this provision being determined no longer by “the text of the law plus technical facts,” but rather shaped by multi-party contestation.

Firstly, the ambiguity of the *EPC* text has given rise to conflicting interpretations. Article 53(b) of the *EPC* contains two key ambiguities: firstly, the scope of animal and plant varieties is not clearly defined; secondly, whilst it excludes “essentially biological production methods”, it does not explicitly state whether “obtaining a product” is patentable, which is the root cause of subsequent interpretative vacillation. As the EPO’s highest judicial body, the EBA’s interpretations should, in principle, be final; however, Decisions G 2/12 and G 2/13 faced immediate counter-pressure from the Administrative Council upon their publication. The Council’s decision to pursue the procedural route of “amending the Rules of Procedure” rather than “amending the Convention” constituted the institutional starting point for the first round of negotiations regarding Article 53(b).

Secondly, the European Commission (EC) utilised its interpretation of *the Directive* as a channel for conveying political will. Article 4 of Directive 98/44/EC is highly analogous to Article 53 of the *EPC*, and likewise excludes only “essentially biological processes” as such. However, in November 2016, the EC issued Notice 2016/C 411/03, providing a “clarification of the legislator’s intent” regarding *the Directive*, indicating that the EPO’s interpretation was inconsistent with the intent of *the Directive*. In legal terms, this Notice constitutes an “explanatory notice”; it has no direct legislative force and does not alter the text of the *EPC*, but it has prompted the amendment of Rule 28(2) of the *EPC* Rules, effectively serving as a channel for the EC—acting behind the scenes of the EPO Administrative Council—to express its views and exert influence. The issue lies in the fact that, while the EPO is an institution independent of the EC and holds the supreme authority to interpret the *EPC*, the EC nevertheless uses amendments to the Rules as a means to influence the interpretation of the *EPC*. This is the subject of the so-called debate over whether “the power of interpretation lies in Brussels (the EC) rather than Munich (the EPO)”.

Finally, the launch of the Unified Patent Court (UPC) system has further fragmented an already dispersed interpretive landscape. Since the UPC became operational in June 2023, the question of interpretive authority under the *European Patent Convention (EPC)* has once again come to the fore. In Decision G 3/19, although the EPO did not directly invoke the judgments of the Court of Justice of the European Union (CJEU) in Cases C-140/12 and C-176/13, it employed a dynamic interpretation approach that brought its substantive conclusions into alignment with the CJEU’s position, while maintaining formal independence in its interpretive authority. This posture of “substantive convergence with formal independence” has sown the seeds for future institutional uncertainty. More notably, under the institutional linkage between the Unified Patent Court Agreement (UPCA) and the CJEU, the UPC may, through the CJEU’s preliminary ruling procedure, extend the scope of the “essentially biological processes” exclusion to new genomic techniques (NGTs) varieties on a case-by-case basis. Should this pathway be activated, the fragile balance established by G 3/19 could be undermined. At present, the debate over the patentability of NGT varieties remains deadlocked between the European Parliament and the Council of Europe, with no final decision yet reached. Once the legislative position is finalised, the EPO’s relevant interpretation standards are likely to require further adjustment.

In summary, the complex misalignment regarding interpretative authority and the ongoing power struggles within the European animal patent protection system have yet to be resolved. The EPO’s G 3/19 interpretation currently rests on a fragile balance, presenting a complex picture of the EU’s animal variety patent protection system as it seeks equilibrium through dynamic adaptation. It is worth noting that some EU Member States have long since introduced a system of variety rights in the field of animal varieties; for example, countries such as Bulgaria have enacted domestic legislation establishing a specialised certificate system for animal varieties similar to that for plant varieties. Although this divergence in Member State legislation does not contravene the EU’s unified policy, it reflects institutional fragmentation within the EU regarding the protection of animal varieties. This is bound to undermine the legal uniformity of the internal market and create institutional barriers to cross-border collaboration on breeding innovation. This divergence also provides empirical evidence from within the EU to support the subsequent discussion on “the feasibility of a dedicated rights approach”.

### 3.2. The Dual Dilemma of the Instrumentalisation of “Dynamic Interpretation” and the Ambiguity of Technical Standards

The dilemmas in the EU’s practice of animal patent protection are epitomised by two interrelated issues: firstly, the oscillation between the “dynamic interpretation” approach in G 2/12 and G 3/19 reveals its inherent lack of internal constraints; secondly, the criteria for distinguishing between “substantive biological processes” and “technical processes” are difficult to apply in the face of the realities of composite breeding techniques, making it challenging to apply the exclusionary boundaries of Article 53(b) consistently.

At the legal theory level, the two-way instrumentalisation of dynamic interpretation erodes legal stability. The EBA introduced the “dynamic interpretation” methodology in G 2/12, asserting that the meaning of a provision may evolve over time. While this approach was used in G 2/12 to exempt products from the scope of the exclusion, five years later in G 3/19 it was applied in the opposite direction to re-exclude them. This ‘both ways’ characteristic indicates that dynamic interpretation lacks an intrinsic constraint mechanism and cannot prevent users from adjusting conclusions according to political winds rather than legal-doctrinal logic. As Cockbain pointed out, following G 3/19, the actual boundaries of Article 53(b) of the *EPC* are no longer the result of the EBA’s purely legal reasoning, but rather a mixture of the political will of the Contracting Parties to the Administrative Council and the judicial guise of dynamic interpretation [34]. The damage this instability inflicts on the EU principle of “Rechtssicherheit” cannot be overlooked: the fact that the boundaries of patentability can be adjusted according to political trends not only increases the costs of patent portfolio management for businesses but also reduces the predictability of the system, placing institutional pressure on small and medium-sized breeders [39].

At the level of examination practice, the criteria for distinguishing between “technical processes” and “biological processes” remain ambiguous. According to the *EPC* and Decision G 3/19, processes relying entirely on substantive biological processes are not patentable, whereas products obtained through decisive modifications, such as those achieved by genetic engineering, may constitute a patentable “technical contribution”. Notably, in recent revisions to its Examination Guidelines, the EPO has introduced requirements for assessing technical contributions, emphasising that the determination of whether a technical step plays a central and irreplaceable role should be based on the “essence of the process” rather than the final result [40]. However, in practice, it is difficult to establish stable and workable application criteria. Modern animal breeding increasingly follows a “composite breeding” model, in which multiple technical pathways—including traditional selection, molecular marker-assisted selection and gene editing—are deeply intertwined. For example, animal individuals in which deleterious genes have been knocked out via gene editing typically still require several generations of traditional crossbreeding and backcrossing to stabilise the traits; in this process, biological selection and technical intervention jointly constitute the mechanism underlying the formation of the final result. The EPO has not yet established a clear framework for the quantitative assessment of multiple factors, and examination decisions remain, to a considerable extent, dependent on the case-by-case judgements of examiners and appeal bodies. More crucially, the patent system struggles to directly cover the final form of the outcome—namely, animal ‘varieties’—forcing applicants to resort to complex claim drafting to circumvent the scope of exclusion.

The instrumentalisation of dynamic interpretation and the ambiguity of examination standards are not isolated phenomena; rather, they are mutually reinforcing symbiotic relationship. On the one hand, the semantic openness of the core concept of “essential biological processes” allows dynamic interpretation to be applied in both directions; on the other hand, the “dual-purpose” nature of dynamic interpretation in turn exacerbates the unpredictability of examination standards. Examiners must not only assess the “core role” of technical steps but also anticipate whether shifting political tides might lead to further adjustments to the exclusion boundaries under the EBA. Taken together, this two intertwined dilemma reveals the EBA’s retreat as a judicial mechanism in the face of political pressure from the administrative sphere, as well as the interpretative inadequacy of the 1973 text of Article 53(b) of the *EPC* when confronted with the realities of composite breeding technologies in the 2020s.

### 3.3. The Flexibility and Paradoxes of Ethical Review as a Constraining Mechanism

If the ambiguity of the exclusion rule in Article 53(b) constitutes a technical dilemma, then the ethical and public order review established by Article 53(a) introduces a deeper level of uncertainty at the level of value judgements.

#### 3.3.1. The Dilemma of Inconsistent Interpretations of the Balancing Test and Reflections Thereon

In the “Harvard tumour-bearing mice” case, the EPO established the “utilitarian balancing test”, noting that the development of new technologies is usually accompanied by risks; however, this should not negate the new technology, but rather require a careful weighing of risks against benefits [41]. In that case, the scientific value of cancer research was deemed to outweigh the ethical concerns regarding animal suffering, and the patent was granted. However, in the subsequent “Upjohn Bald Mice” case, the same test led to the opposite conclusion: the genetic modification, intended to test a product for treating human baldness, subjected the animals to unnecessary suffering, and its benefits were insufficient to offset the ethical costs [42].

Whilst much of the existing literature regards the comparison of these two cases as emblematic of the principle of balancing interests, few have questioned why the same set of assessment tools yielded opposite conclusions in structurally similar cases. The two cases exhibit a high degree of isomorphism in the following dimensions: (1) both involve genetic modification of non-human mammals; (2) the modifications result in the animals bearing an additional physiological burden; (3) the purpose of the modifications is to serve human health needs; (4) in both cases, there were no viable non-animal alternatives. The main distinction between the two cases lies in the gradations of “scale of the beneficiary population” and “severity of the disease”; however, it is precisely these gradations—rather than any principled ethical distinction—that led to diametrically opposed examination conclusions. This demonstrates that the operationalisation of the utilitarian balancing test relies heavily on the reviewer’s subjective valuation of “benefits” and “costs”, yet the EPO has never established a transparent set of criteria for assessing value and ethics [43]. This shortcoming raises fundamental questions regarding the reliability of ethical review as a governance tool; if the same testing methodology cannot guarantee consistent conclusions when applied to structurally similar cases, the very foundation of its legitimacy as an institutionalised review tool is already undermined.

The flexibility of ethical review has led to three consequences. Firstly, it has opened up scope for regulatory arbitrage, whereby applicants can strategically select technical pathways, claim wording and embodiment designs to steer examiners towards favourable ethical judgements. For instance, in the “Upjohn hairless mouse” case, had the applicant reframed the invention’s purpose as “research into the fundamental biological mechanisms of hair follicle development”, the “scientific value” attributed to it might have been higher, thereby undermining the substantive effect of the ethical review [6]. Secondly, the choice of location for R&D investment is distorted. When the EU’s ethical review standards are stricter and more unpredictable than those of other jurisdictions, biotechnology companies may shift to jurisdictions with more lenient review processes; this is particularly pronounced in cutting-edge research fields. Thirdly, compliance costs for small and medium-sized entities are disproportionately driven up—while large multinational corporations have the capacity to monitor developments in ethical review and mitigate risks through meticulous claim drafting, small and medium-sized breeding entities lack the corresponding resources, creating a technical barrier that undermines the diversity of the innovation ecosystem [44].

#### 3.3.2. The Logical Transition from the “Assumption of Commensurability” to the “Value No-Go Zone”

If the failure of the utilitarian balancing test stems from its implicit assumption that “human benefits and animal welfare are commensurable”, then the “value no-go zone model” represented by T 1553/22 (the human–pig chimaera case) marks a complete shift in ethical review standards.

Signs of this shift had long been apparent; in its 2008 decision G 2/06, the EBA stated that “if the implementation of an invention necessarily involves the destruction of human embryos, its commercial exploitation would be contrary to public order and morality, even if the invention were of significant medical value.” [45]. Although this case was limited to the scenario of “embryo destruction”, it may be regarded as an early manifestation of the “value-based exclusion” model. Case T 1441/13 further explicitly recognised that “human dignity” may serve as a ground for exclusion independent of utilitarian calculations [46]. In Case T 1553/22, the EBA explicitly stated that if there is a realistic possibility of human cells integrating into the brain or germline of a human–animal chimaera, patentability should be excluded on the grounds of an affront to human dignity; even significant therapeutic benefits cannot override this [47]. Under the “value-based exclusion model”, certain types of inventions are pre-excluded and are no longer subject to utilitarian calculations of benefits versus harms. This shift acknowledges that certain ethical values, such as human dignity, are incommensurable and should not, nor can they, be subjected to a balancing of interests. In this regard, the revision history of the *Examination Guidelines* serves as institutional evidence: the 2014 edition incorporated the “embryo destruction” exclusion into G 2/06; the 2019 edition added warnings regarding “human germline editing” and “human-animal chimeras”; and the 2024 edition provided even stricter exclusion guidelines [48].

In summary, T 1553/22 is not an isolated instance of paradigm shift in ethical governance, but rather a direct institutional response to the failure of the utilitarian balancing test. When the “assumption of commensurability” fails to yield consistent outcomes on a case-by-case basis, the examining authority turns to delineating absolute prohibitions, so as to prevent value conflicts from recurring in similar cases.

This shift, however, has not fundamentally resolved the issue of the reliability of governance instruments; it has merely transformed the nature of uncertainty from “inconsistency in the outcomes of balancing” to “lack of clarity in the boundaries of the prohibited zones”. More specifically, how should the concept of “human dignity” be defined? Can “significant therapeutic benefits” under no circumstances justify crossing the prohibited boundaries? These questions remain to be answered. It is thus evident that the predictability of the ethical review mechanism has not been substantially improved by this transformation.

## 4. Strategies for Establishing a System for the Protection of Intellectual Property Rights in Animal Varieties in China: Lessons from the European Union

### 4.1. The Basis and Necessity of Drawing Lessons from the EU’s Animal Patent Protection

When exploring pathways to improve China’s intellectual property protection system for animal varieties, the European Union constitutes the most direct and irreplaceable point of reference due to the isomorphism of its institutional framework with China’s and the common origins of their challenges.

Firstly, China and the EU exhibit significant institutional isomorphism in the field of animal patent protection. From the perspective of legislative architecture, China’s institutional arrangement—based on the *Patent Law* and with the National Intellectual Property Administration as the examining authority—is highly consistent with the EU’s approach, which is based on the *EPC* and with the EPO as the examining authority. Article 25 of the *Patent Law*, which states that “no patent shall be granted for animal varieties”, corresponds to Article 53(b) of the *EPC*; Section 4 and Section 5 of Chapter 1, Part II of the *Examination Guidelines* correspond to the ethical exclusions under Article 53(a) of the *EPC*. This dual-track structure of “technical exclusions plus ethical exclusions” is itself a direct transposition of the provisions of Article 53 of the *EPC*.

By contrast, countries such as the United States and Japan fall within an “institutional heterogeneity” framework. The United States adopts a model of broad-scope patent examination coupled with separate regulatory oversight by the FDA and IACUC; Article 32 of *The Japanese Patent Act* does not expressly exclude animal varieties. These countries adopt a dual-track approach of patent protection for methods and products, coupled with trade secret protection for bred populations. Whilst this avoids the interpretative difficulties associated with subject-matter exclusion clauses, it faces issues such as the fact that the varieties themselves can only be indirectly protected through claims for “product of a process” or “genetic characteristics”, the extreme complexity of claim drafting, and difficulties in the circulation of germplasm due to trade secret protections. More importantly, the US and Japanese models lack the institutional foundation of a “subject-matter exclusion clause”—a feature shared by China and the EU—and are therefore unable to provide China with direct experience on “how to find scope for protection within the statutory exclusion framework”; this is precisely where the EU’s value as a model lies.

Secondly, China and the EU face highly analogous practical issues. First, the problem of the “dynamic interpretation” of exclusion clauses also exists in China. The term “varieties” in Article 25 of the *Patent Law* shares the same semantic openness as “animal varieties” in Article 53(b) of the *EPC*. The CNIPA’s *Examination Guidelines* interpret “animal varieties” as “including their germ cells, embryos, etc.”, which in itself constitutes an expansive interpretation. Lessons from the EU demonstrate that once dynamic interpretation is influenced by shifting political or ethical trends, exclusion clauses can swing in either direction; this offers practical guidance for the future judicial interpretation of Article 25 in China. Second, there is the issue of predictability regarding the integration of ethical review into patent procedures. Article 38 of *China’s Biosafety Law (2021 edition)* and Article 15 of the *Livestock Law (2023 edition)* stipulate, respectively, that gene-edited animals require ethical review and that genetically modified livestock and poultry require safety assessments and ethical review. This forms a triple filtering mechanism comprising “patent examination + biosafety + ethics”, which is highly analogous in structure to Article 53(a)(b) of the *EPC* combined with the EU Biosafety Directive. The 2023 “Patent Examination Guidelines” have added examples of “contravention of public morality”, incorporating animal gene editing and human–animal chimaeras into the scope of precautionary exclusions, in line with the EU’s transition towards a new ethical governance paradigm.

Thirdly, there is a practical necessity for China to improve the protection of intellectual property rights for animal varieties. First, this is an intrinsic requirement for incentivising breeding innovation against the backdrop of the revitalisation of the seed industry. The research and development of new animal varieties involves long cycles, substantial investment and results that are easily disseminated. Currently, China’s self-sufficiency rate for livestock, poultry and aquaculture breeding stock is steadily increasing, yet capacity for high-end breeding innovation remains insufficient. However, the *Patent Law* provides protection only for “production methods”; whilst the 2025 *Patent Examination Guidelines* clarified the definition of “plant varieties” and aligned it with the *Seed Law*, this logic has not been extended to the animal sector [14]. Second, the administrative management model centred on the *Livestock Law* has functional limitations. Although the law stipulates that “legitimate rights and interests are protected by law”, it does not employ property rights terminology such as “special rights” or “monopoly rights” [48]. In practice, new animal varieties rely primarily on administrative approval by the agricultural authorities; this essentially constitutes an administrative licence for market access, rather than a property rights system conferring special rights on breeders. Judicial practice reveals that breeders who have obtained approval certificates find it difficult to assert prior rights or bring infringement proceedings when varieties with highly similar traits are approved [49]. This systemic constraint has been corroborated from multiple sources: at the policy level, the *Action Plan for the Revitalisation of the Seed Industry* acknowledges the need to “improve the intellectual property system” and the “insufficient incentives for breeding innovation” [50]; at the industrial level, Chen Yaosheng, Chief Scientist of the National Swine Industry Technology System, points out that the breeding cycle for core swine herds lasts as long as 8–10 years, and without exclusive variety rights protection, enterprises find it difficult to bear the sunk costs of the initial screening phase [51]. Third, from the perspective of industrial competition, establishing a dedicated protection system is a strategic necessity for enhancing the international competitiveness of China’s seed industry. The Ministry of Agriculture and Rural Affairs has repeatedly emphasised the need to “strengthen intellectual property protection in the seed industry” and “cultivate and expand leading seed enterprises” at meetings on the revitalisation of the seed industry, which clearly reflects an awareness of this institutional shortcoming [50]. Although this policy is primarily aimed at plant varieties, its rationale is equally applicable to the field of animal breeding.

In summary, the value of the EU as a model for China’s intellectual property protection system for animal varieties is rooted in the high degree of similarity between the two in terms of institutional foundations, problem structures and policy challenges, as well as the pressing practical need. Countries such as the US and Japan offer valuable reference points at the level of technical alignment, but cannot replace the direct lessons provided by the EU regarding “interpretation risks associated with subject-matter exclusions” and “the integration of ethical review into patent procedures”. For China, which is currently at a critical stage of institutional development, the institutional costs of correcting errors arising from a misguided interpretative approach would far exceed those of prudent design at the outset.

### 4.2. Pathway Selection and Addressing Tensions in China’s Animal Variety Protection System

The preceding discussion has indicated that the EU’s experience holds unique value as a model for China. However, a review of the EU’s institutional evolution also reveals that incremental reform is insufficient to resolve the underlying issues. From G 2/12, which employed dynamic interpretation to exempt products from the scope of exclusion, to the administrative countermeasures embodied in Rule 28(2), and finally to G 3/19, which reimposed the exclusion, each adjustment has merely relocated the problem rather than fundamentally resolving the structural tension between patent protection and animal variety protection. The parallel shift in ethical review—from a “utilitarian balance” to a “value-based exclusion model”—has further entrenched institutional uncertainty. The EU’s experience thus demonstrates that, without altering the fundamental architecture of the patent system, reliance on judicial interpretations, amendments to implementing regulations, and adjustments to examination guidelines cannot provide stable and predictable protection for animal breeding.

At a deeper level, this is rooted in a structural tension between the patent system and the protection of innovation in animal breeding—a tension that cannot be reconciled through incremental reform. The first dimension of this tension concerns the objects of protection. The patent system protects ‘technical solutions’, whereas the core output of animal breeding is the “variety” itself—a holistic genetic resource pool formed through multiple generations of selection. Claims drafted in “product-by-process” form, which seek to indirectly encompass varieties, inevitably reduce holistic biological achievements to a fragmented collection of technical features; this approach neither provides adequate protection for the variety nor yields claims with clear and predictable boundaries. The second dimension concerns the standards for grant. The patent law’s “inventive step” requirement demands substantive innovation, whereas the logic of variety protection is to encourage the accumulation and dissemination of superior genetic resources. Traditional hybrid breeding varieties may not satisfy the patent law’s inventiveness threshold, while gene-edited products may be denied “variety” status for failing to comply with DUS criteria. Imposing patent standards on breeding achievements would thus produce a dilemma: either excluding a substantial number of valuable traditional breeding outcomes, or lowering the inventive step threshold to the point where the patent system’s effectiveness is undermined.

This structural tension has become fully apparent in the European Union, yet the limitations of China’s incremental reform approach are even more pronounced. Unlike Article 53(b) of the *EPC*, which remains open to adjustment through dynamic interpretation, Article 25 of China’s *Patent Law* establishes a statutory exclusion; any amendment thereto requires legislation by the Standing Committee of the National People’s Congress. Nor could the CNIPA transcend the textual limits of Article 25 through an expansive interpretation of the term “variety” in the Examination Guidelines. It follows that China should not simply expand the scope of patent protection for animal varieties, but should instead develop an institutional framework that integrates patent protection for technical inventions with a dedicated system for variety protection. This would entail, on the one hand, continuing to refine the patent system’s coverage of breeding-related technical inventions, and on the other, establishing “animal variety rights” in parallel with plant variety rights through the enactment of a dedicated *Animal variety Rights Law*.

However, the creation of a new variety rights system requires resolving issues including overlapping protection, regulatory burdens, and inter-agency coordination; relevant issues must be addressed and solutions discussed. There are three main aspects.

Firstly, overlapping protection and blurred boundaries, and how to address them. If animal variety rights are introduced alongside the patent system, the same breeding achievement may fall under both types of protection simultaneously. The accumulation of variety rights licence fees and patent licence fees will increase costs for downstream users; the breeder’s exemption under the variety rights system, for which no corresponding exemption exists under patent law, may also give rise to conflicts of rights. The institutional design to address this issue may proceed along two levels. At the legislative level, a foundational demarcation of subject matter should be established—clarifying that the object of protection under the animal variety rights system is the animal variety itself, provided it meets the DUS criteria, while breeding techniques and methods remain governed by the *Patent Law*. At the level of rights exercise, a restrictive provision should be introduced, specifying that patent holders may not rely on their patents to prevent variety rights holders from exercising the breeder’s exemption.

Secondly, the barriers to entry for small and medium-sized breeders and how to address them. Given that livestock and poultry breeding in China is predominantly carried out by small and medium-sized enterprises (SMEs) and local conservation centres, if the costs of applying for plant variety rights are significantly higher than the expected returns, the system may instead constitute a ‘regulatory barrier to entry’. In 2024, a survey of European SMEs conducted by Technopolis has already shown that the combination of patents and cumulative licence fees exposes SMEs to the risk of “unintended infringement”, with institutional costs being internalised by large companies whilst small enterprises bear the burden [52]. This issue can be addressed by waiving or reducing application and maintenance fees for small and medium-sized breeding entities, local conservation centres, and research institutes, while encouraging provincial agricultural and rural affairs authorities to establish guidance centres for variety rights applications, so as to lower the information barriers associated with agency services.

Thirdly, there is the dispute over definitions between the Patent Office and the plant variety rights authority, and how to address it. The same term, “animal variety”, has different meanings under different sets of rules, which may result in a two-way failure of the protection system. In this regard, it is recommended that the boundary between the scope of exclusion under Article 25 of the *Patent Law* and the objects of protection under the *Animal Variety Rights Law* be clarified: variety rights protect animal varieties that meet the DUS criteria, while breeding intermediates, genetic constructs and the like are governed by the *Patent Law*. Specific definitions may be clarified through departmental regulations jointly issued by the Ministry of Agriculture and Rural Affairs and the CNIPA.

### 4.3. Proposal for the Establishment of an Animal Variety Rights System

The framework for the animal variety rights system may be designed adaptively by drawing on the experience of plant variety protection whilst taking into account the specific characteristics of animal breeding. The institutional framework should focus on the following three aspects.

Firstly, establishing differentiated authorisation criteria. Plant variety rights are based on the core authorisation criteria of Distinctness, Uniformity and Stability (DUS). However, the biological characteristics of animal breeding differ significantly from those of plants: animals reproduce in family lines, exhibit sexual dimorphism, have population sizes constrained by breeding cycles, and gene editing can produce decisive changes at a single locus. Furthermore, the outcomes of traditional hybrid selection and NBT editing—the two main technical approaches—differ in form, necessitating differentiated adjustments to the DUS criteria.

With regard to novelty, the general rules of the UPOV Convention serve as an appropriate reference: “not having been sold within the territory for more than one year, or sold abroad for more than three years, prior to the filing date”. Given that animal breeding cycles are typically longer than those for plants (the generation interval for cattle, for instance, is approximately 4–5 years), and that the commercialisation of animal varieties frequently commences with the limited distribution of a core herd, this paper proposes a grace period of one year for domestic sales and three years for overseas sales. These figures are extrapolated by analogy from the plant variety protection framework and should not be regarded as empirically established thresholds. To that end, the Ministry of Agriculture and Rural Affairs, in consultation with breeding industry associations, should undertake species-specific studies on the reproductive cycles of different livestock categories, with a view to establishing differentiated thresholds in future legislation.

The requirement for distinctiveness entails significant superiority over the parental population or control variety in at least one economic trait. For traditionally bred varieties, distinctiveness stems from the cumulative effects of multiple genes; examination may rely on pedigree records and inter-farm performance testing. For gene-edited varieties, distinctiveness may result from targeted modification at a single locus; the assessment should shift from “phenotypic differences in traits” to “the association and robustness between the target locus and the phenotype”. *Japan’s Patent Examination Guidelines (2025 edition)* set out differentiation criteria for gene-edited and traditionally bred varieties; its approach of adjusting the focus of examination according to the technological pathway is worthy of reference [53].

With regard to consistency, in light of the biological characteristics of animals, fluctuations in the traits of animal populations within the scope of expected natural variation shall be permitted. For mammals, variations in coat colour within a pedigree and individual body size fluctuations shall be permitted; for poultry, population fluctuations in indicators such as egg production and slaughter weight, measured by standard deviation, shall be permitted; for aquaculture, reasonable batch-to-batch variation in traits shall be permitted. Specific differentiated provisions for different traits shall be set out in the *Implementing Rules of the Animal Variety Rights Law*.

With regard to stability, conventionally bred varieties shall be required to exhibit stable traits over three consecutive generations. This threshold rests on two grounds: first, Article 9 of the *Bulgarian Law on the Protection of Animal Varieties* mandates that stability tests for livestock varieties demonstrate “no significant deviation in observable traits over at least three consecutive generations”; second, according to the theoretical predictions of quantitative genetics, the genetic structure of a population stabilises from the F_3_ generation onward following multiple generations of selective mating, with trait variance tending to converge—at which point stability assessments attain a high degree of reliability [54]. For gene-edited varieties, the conventional “three-generation stability” criterion is inapplicable; rather, it must be verified that the strong association between the target gene locus and the phenotype persists across generations—namely, that the edited locus remains stable, off-target effects are controlled, and the background genome exhibits no significant unintended drift. The above threshold shall serve as a legislative benchmark, to be adjusted on a species-specific basis through technical guidelines that take due account of the generational intervals and breeding methods of each livestock category.

Admittedly, while the above authorisation criteria draw upon prior experience in the plant sector, they still require appropriate adaptation for the animal sector. One issue that warrants discussion is that, just as the system of Essential Derived Variety (EDV) was established in the plant sector to curb imitation breeding, similar challenges may arise in the animal sector—particularly with respect to gene-edited animals. Drawing on the practical experience of the EDV system, this paper proposes that original innovation in breeding can be incentivised by raising the authorisation examination standards—particularly by tightening the requirements for reviewing economic traits under the distinctiveness criterion. In addition, innovative authorisation mechanisms such as open licencing could be introduced to mitigate interest disputes between prior and subsequent breeders. Nevertheless, the distinctive nature of breeding dictates that the issue of derived varieties is unavoidable. In the future, the allocation of interests between prior and subsequent breeders will still need to be assessed and discussed on a case-by-case basis in specific practices.

Secondly, there are conditions for refusal of authorisation. The subject matter of variety rights is a “population of living animals”; the authorisation decision implicitly recognises that the variety may be “legally bred and sold”, and therefore ethical and ecological boundaries shall be embedded as prerequisites. Drawing on existing Chinese regulations and EU practices, the following three categories of cases shall be explicitly excluded from authorisation: (1) Human–animal chimaeras: animals containing human gene sequences that lack significant medical or scientific value (such as the implantation of human neural progenitor cells into a pig’s brain solely for ornamental purposes or non-essential ‘proof-of-concept’ research); such varieties, which offend against human dignity, shall be excluded; (2) Species posing ecological risks: those that may transmit zoonotic diseases (such as edited aquatic species with enhanced disease resistance but which become viral reservoirs) or disrupt the stability of local food chains (such as non-native edited species that, if they escape, pose a risk of genetic introgression or predation pressure on indigenous species); authorisation must be subject to a joint review by the ecological and environmental authorities prior to approval; (3) Violations of animal rights: Authorisation shall not be granted for genetic modifications that may result in sustained impairment of an animal’s basic survival capabilities and which have no clear medical or industrial value. Examples include chronic skeletal lesions caused by genetic editing aimed at achieving “dwarf ornamental traits”, and skin barrier defects resulting from editing for “fur texture”. Such modifications that cause animal suffering for no purpose other than satisfying human aesthetic or commercial decorative purposes can be intercepted at the stage of variety rights authorisation.

Thirdly, the scope and limitations of rights. Drawing on the UPOV framework, holders of animal variety rights shall enjoy exclusive rights to the production, propagation, sale, offer for sale, import, and export of propagating material of authorised varieties (including breeding livestock, breeding poultry, fish fry, frozen semen, and embryos). At the same time, reasonable limitations on these rights must be established to balance the public interest. Three specific limitations should be clearly defined:

Scientific research exemption: Basic research does not in itself create direct commercial competition; excessive exclusivity would, in fact, hinder subsequent innovation. The use of authorised varieties for scientific breeding purposes should be permitted, including their use as parental stock for subsequent breeding operations such as hybridisation, mutagenesis, and genetic editing.

Farmers’ privilege: Farmers shall be permitted to retain and use the offspring of protected animals for agricultural production on a non-commercial scale (e.g., a dairy farm retaining heifers to replenish its herd), but shall not be permitted to propagate them on a large scale for commercial sale. The threshold for “non-commercial scale” shall be set differently according to livestock species (e.g., for pigs, “self-bred and self-used with an annual slaughter of 50 head or fewer”; for dairy cattle, “retaining heifer calves from a herd of 10 head or fewer”).

Compulsory licencing: A compulsory licencing system shall be established in the national or public interest (e.g., the supply of vaccine seed stock during major animal disease outbreaks, or the emergency conservation of genetic resources). Licence fees shall be determined in accordance with the standard of “reasonable compensation” and shall be subject to the approval of the Ministry of Agriculture and Rural Affairs in conjunction with the competent pricing authorities.

### 4.4. Embedding Ethical Review Safeguards

The breeding and commercial utilisation of animal varieties involve profound value issues such as animal welfare, ecological security, and public morality; therefore, ethical review must be deeply embedded within the institutional design.

To avoid the overly broad application of ethical review, its scope and applicable subjects should be clearly defined. Ethical review should focus on breeding types and technical approaches that may pose significant ethical risks, particularly those involving genetic engineering or other improvement activities that have a substantial impact on the physiological structure or behavioural capacity of animals.

With regard to the scope of application, an approach combining “principled coverage with specific enumeration” could be adopted: a general provision should be established stipulating that any new variety likely to have a significant impact on animal welfare, health, or basic behavioural capabilities shall undergo ethical review, while an annex should set out specific provisions listing high-risk breeding scenarios.

With regard to specific criteria, the animal variety rights system is, in essence, a mechanism for the recognition of rights rather than for market promotion; furthermore, China already has a well-established approval system. Therefore, ethical review should serve as a baseline safeguard rather than a stringent prior approval process. It should thus be stipulated that, except where there is clear scientific significance, potential for medical application, or a significant public interest objective, the breeding of animal varieties shall not cause unnecessary suffering, persistent harm, or a significant reduction in the animals’ viability.

For high-risk breeding improvements, China’s ethical review authorities should conduct a substantive review of the *Animal Welfare Impact Statement* submitted by breeders. During the review process, a multi-dimensional indicator system—covering aspects such as the expression of natural behaviours, disease incidence, and effects on lifespan—may be established to assess the animals’ quality of life.

The introduction of this Statement draws upon the relevant mechanisms of the United Kingdom’s *Genetic Technology (Precision Breeding) Act* 2023. Under that Act, gene-edited animals are required to undergo a welfare impact assessment by an independent welfare advisory body before they can receive commercialisation approval.

The implementation of this mechanism in the UK provides preliminary empirical evidence of its effectiveness. According to assessment reports published in recent years by the UK Department for Environment, Food and Rural Affairs (Defra), the involvement of the welfare advisory body has effectively achieved the Act’s objectives of safeguarding animal health and welfare and promoting sustainable development [55]. This indicates that ex ante welfare assessments can effectively mitigate animal welfare risks without constituting an unacceptable barrier to technological innovation.

However, this mechanism also has several limitations that China should be mindful of in its institutional design. For example, the independence of the advisory body has been questioned (its members are appointed by Defra, with some coming from industry); the assessment criteria lack quantitative indicators, resulting in insufficient comparability between cases; and the assessment conclusions are not legally binding, as the competent authorities retain the discretion to grant approval despite dissenting opinions.

These limitations offer the following insights for China. First, the assessing body should ensure its independence; it is recommended that an independently operated National Animal Ethics Review Centre be established under the National Committee for the Review of New Animal varieties, comprising experts in fields such as genetics, animal science, law, and ethics, as well as representatives from animal welfare organisations, and implementing a term-of-office system and a conflict-of-interest avoidance mechanism. Second, assessment criteria should be quantified as far as possible; welfare risk assessment matrices applicable to different livestock species could be developed (such as weighted scoring for indicators including duration of pain, degree of behavioural restriction, and mortality rate). Third, assessment conclusions should carry substantive binding force, and “high-risk” varieties shall directly trigger conditions for refusal of authorisation.

With regard to the review process, a coordinated mechanism combining prior review and dynamic supervision should be established. Applicants shall be required to make the necessary disclosures regarding breeding technical approaches, potential ethical risks, and risk mitigation measures, and to make core review conclusions and regulatory information publicly available, provided this does not involve trade secrets. For breeders who breach ethical review obligations, conceal material information, or circumvent the review process, multi-tiered legal consequences shall be stipulated, including revocation of rights, administrative penalties, and civil liability; where ethical review conclusions are manifestly inappropriate, the relevant parties shall be granted the right to apply for a review or seek redress, in order to prevent the abuse of review powers. During the term of the variety rights, a dynamic regulatory mechanism should be established to continuously assess the impact on animal welfare; where necessary, procedures for restricting, suspending, or even revoking authorisation shall be initiated in accordance with the law. By embedding the assessment of the impact on animal welfare throughout the entire process of granting and exercising new animal variety rights, ethical review will no longer be an external constraint on innovative achievements, but will instead constitute an endogenous element of the institutional design.

## 5. Conclusions

The evolution of the EU’s animal variety patent protection system reveals the structural limitations of the patent system—demonstrating that, without altering its fundamental framework, neither dynamic interpretation nor amendments to implementing rules can provide stable protection for animal breeding. At the same time, the instability and blurred boundaries of ethical review further expose the institutional costs of addressing ethical issues within the patent framework. This paper presents three key findings:

First, the root cause of the EU’s dilemma lies not in ambiguous technical standards or intense political manoeuvring, but in a structural contradiction at the “institutional-genetic level”—while the patent system remains effective in incentivising upstream technological innovation, it cannot adequately protect breeding achievements through patents.

Second, Article 25 of China’s *Patent Law* explicitly excludes “animal varieties” and “plant varieties”, and its rigid constraints leave even less room for incremental reform than in the EU. It is difficult to adopt an expansive interpretation of “varieties”, and it is not possible to create additional scope for protection outside the statutory exclusions. Any interpretative attempt that seeks to push beyond the boundaries of this provision would likely face legal challenges on the ground that it contravenes the mandatory provisions of the *Patent Law*.

Third, establishing a dedicated animal variety rights system does not negate the role of the patent system in protecting breeding-related technical inventions; rather, it advocates replacing the path dependency of incremental patent amendments with a layered approach in which “technical patents protect methods, and variety rights protect outcomes”. Any potential tensions arising from this arrangement can be resolved through institutional design.

China now faces an institutional opportunity that the EU has never had: building upon the mature experience of plant variety rights, it can, in this institutional vacuum, directly establish an animal variety rights system that is coordinated with patents for breeding technologies. This system should include differentiated authorisation criteria, negative thresholds embedded with ethical safeguards, and a mechanism for limiting rights that incorporates a breeder’s exemption. This endeavour not only transcends the EU’s institutional experience but also represents a significant step forward in exploring China’s path to biotechnology governance.

## Data Availability

The data are available upon request from the corresponding author.
